# Differential methylation patterns in lean and obese non-alcoholic steatohepatitis-associated hepatocellular carcinoma

**DOI:** 10.1186/s12885-022-10389-7

**Published:** 2022-12-06

**Authors:** Emma Hymel, Kurt W. Fisher, Paraskevi A. Farazi

**Affiliations:** 1 Department of Epidemiology, University of Nebraska Medical Center, 984395 Nebraska Medical Center, Omaha, NE 68198-4395 USA; 2 Department of Pathology and Microbiology, University of Nebraska Medical Center, Omaha, NE USA

**Keywords:** Liver cancer, Non-alcoholic fatty liver disease, Methylation

## Abstract

**Background:**

Nonalcoholic fatty liver disease affects about 24% of the world’s population and may progress to nonalcoholic steatohepatitis (NASH), cirrhosis, and hepatocellular carcinoma (HCC). While more common in those that are obese, NASH-HCC can develop in lean individuals. The mechanisms by which HCC develops and the role of epigenetic changes in the context of obesity and normal weight are not well understood.

**Methods:**

In this study, we used previously generated mouse models of lean and obese HCC using a choline deficient/high trans-fat/fructose/cholesterol diet and a choline supplemented/high trans-fat/fructose/cholesterol diet, respectively, to evaluate methylation differences in HCC progression in lean versus obese mice. Differentially methylated regions were determined using reduced representation bisulfite sequencing.

**Results:**

A larger number of differentially methylated regions (DMRs) were seen in NASH-HCC progression in the obese mice compared to the non-obese mice. No overlap existed in the DMRs with the largest methylation differences between the two models. In lean NASH-HCC, methylation differences were seen in genes involved with cancer progression and prognosis (including HCC), such as CHCHD2, FSCN1, and ZDHHC12, and lipid metabolism, including PNPLA6 and LDLRAP1. In obese NASH- HCC, methylation differences were seen in genes known to be associated with HCC, including RNF217, GJA8, PTPRE, PSAPL1, and LRRC8D. Genes involved in Wnt-signaling pathways were enriched in hypomethylated DMRs in the obese NASH-HCC.

**Conclusions:**

These data suggest that differential methylation may play a role in hepatocarcinogenesis in lean versus obese NASH. Hypomethylation of Wnt signaling pathway-related genes in obese mice may drive progression of HCC, while progression of HCC in lean mice may be driven through other signaling pathways, including lipid metabolism.

**Supplementary Information:**

The online version contains supplementary material available at 10.1186/s12885-022-10389-7.

## Background

Nonalcoholic fatty liver disease (NAFLD) affects approximately 24% of the world’s population [1]. NAFLD encompasses a spectrum of diseases characterized by fat in the liver that may progress to nonalcoholic steatohepatitis (NASH) with inflammation and fibrosis and ultimately to cirrhosis, which results in an increased risk of developing hepatocellular carcinoma (HCC) [2]. Treatment options for advanced stage HCC are limited, so understanding the development of HCC may identify opportunities for drug interventions or opportunities for primary prevention efforts [3]. Knowledge of the role of diet in the development of HCC has great importance for understanding the mechanism by which NASH progresses to HCC.

While often associated with obesity, NAFLD may develop among lean individuals as well, especially among those that are of normal weight but metabolically obese [1]. The exact causes of lean NAFLD are not clear, but those with lean NAFLD are less likely to have obesity-related co-morbidities [4, 5]. The role of diet in the progression of NASH to HCC is not well understood. The complex pathways involved in NASH-related HCC likely involve genetic and epigenetic factors [2]. Differential methylation patterns of HCC may be useful in developing pharmacological interventions, since DNA methylation is reversible and hence susceptible to intervention [6].

It was previously found that the remodeling of DNA methylation occurs at genes in patients with NASH and fibrosis, suggesting that epigenetic signatures may be a possible biomarker for severity of disease [1]. Previous studies have identified potential causal relationships between epigenetic changes and liver carcinogenesis [6]. In HCC, hypomethylation has been found with transcriptional enhancers and hypermethylation has been found with promoter-associated CGIs and cis-regulatory elements [6]. Additionally, lower expression of phosphatidylethanolamine N-methyltransferase (PEMT) was found in individuals with lean NASH, which could be implicated in its progression [5].

To date, only a few studies have investigated the role of epigenetic changes in the progression of NASH-related HCC in lean versus obese individuals. In this study we used previously developed novel models of lean and obese NASH-HCC in mice using choline deficient (CD) and choline supplemented (CS) high trans-fat/fructose/cholesterol diets to examine differences in DNA methylation during the progression of NASH to HCC as well as differences of DNA methylation in HCC progression in lean versus obese mice.

## Methods

### Animals and experimental diets

This study was approved by the Institutional Animal Care and Use Committee at the University of Nebraska Medical Center (Protocol #: 17–018) and was also conducted in compliance with the ARRIVE guidelines**.** Male (*n* = 103) C57BL/6 N mice (Charles River Laboratories) were allowed to acclimate and housed as previously described beginning at 3 weeks of age [7]. 30 males were fed a choline supplemented, high trans-fat, fructose, and cholesterol diet (CS-HFFC; D18091706), 38 males were fed a choline deficient, high trans-fat, fructose, and cholesterol diet (CD-HFFC; D17071001), and 35 males were fed a low-fat control diet (CON; D16120211; Research Diets, New Brunswick, New Jersey). The estimated HCC penetrance from our previous work was used to determine this sample size [7]. The consumption of food was monitored, and the mice were regularly weighed and husbandry checks were performed as previously described [7].

### Histological evaluation

All mice were monitored until the endpoint of the study (64 weeks of age). Any mice showing signs of poor health were euthanized per institutional ethical guidelines by CO_2_ inhalation. After harvesting tissues for analysis, exsanguination was done to confirm death. A cardiac puncture was performed to collect blood right after euthanasia was performed. Livers were excised, weighed, and observed grossly for the appearance of nodules. The tissue samples were snap frozen in liquid nitrogen and then stored at − 80 °C. The remaining tissues were fixed in 10% formalin for 2 hours and paraffin embedded at the Tissue Sciences Facilities at the University of Nebraska Medical Center. Hematoxylin and Eosin (H&E) and Masson-Trichome were used to stain tissue sections. An additional reticulin stain was added at necropsy. An experienced pathologist blindly evaluated the stained sections, scoring the sections for steatosis, ballooning, and inflammation to determine the presence of NAFLD and NASH [8]. The stained liver sections were also evaluated for the presence of regenerative nodules, dysplastic nodules, and hepatocellular carcinomas as previously described [7].

### DNA extraction and RRBS

Forty samples were selected for analysis: normal liver tissue from 6 controls, NASH tissue from 4 CD-HFFC fed mice, dysplastic tissue from 7 CD-HFFC fed mice, HCC tissue from 7 CD-HFFC fed mice, NASH tissue from 2 CS-HFFC fed mice, dysplastic tissue from 8 CS-HFFC fed mice, and HCC tissue from 6 CS-HFFC fed mice. DNA was isolated from 25 mg of snap-frozen liver tissue using the Qiagen DNeasy blood and tissue kit (Qiagen, Germantown, MD). Genome wide DNA methylome analyses were carried out on DNA samples (400-500 ng) using the Diagenode Inc. (Denville, NJ) Premium Reduced Representation Bisulfite Sequencing (RRBS) kit on mouse samples. DNA concentration of the samples was analyzed using the Qubit® dsDNA Assay Kit (Thermo Fisher Scientific) and DNA quality was assessed using the Fragment Analyzer™ and the DNF-488 High Sensitivity genomic DNA Analysis Kit (Agilent).

### DNA methylation data analysis

The output from RRBS was read into the methylKit (v1.16.1) package in R (v4.0.3) and analyzed by the Bioinformatics and Systems Biology Core at the University of Nebraska Medical Center [9]. Logistic regression tests were used for the differential methylation analyses and the sliding linear model (SLIM) method was used for multiple testing adjustment on 15 comparisons [10]. Generated q-values were used for representing the adjusted *p*-values. Significance levels were determined by the criteria q < 0.01 and percent methylation differences larger than 25%.

### Annotation and functional pathway analysis

MethylKit was used to annotate the differentially methylated regions to include the distance to the corresponding gene and gene ID. UniProt and Reference sequence (RefSeq) were used to identify gene names and function for significantly differentially methylated regions [11, 12]. Gene Ontology enrichment analysis was done on the differentially methylated regions between the choline deficient and choline supplemented HCC samples and between HCC and NASH in both models to find the molecular function of regions that are over- or under-represented in the sample [13–15].

## Results

### Characterization of differentially methylated regions

**Fig. 1:**
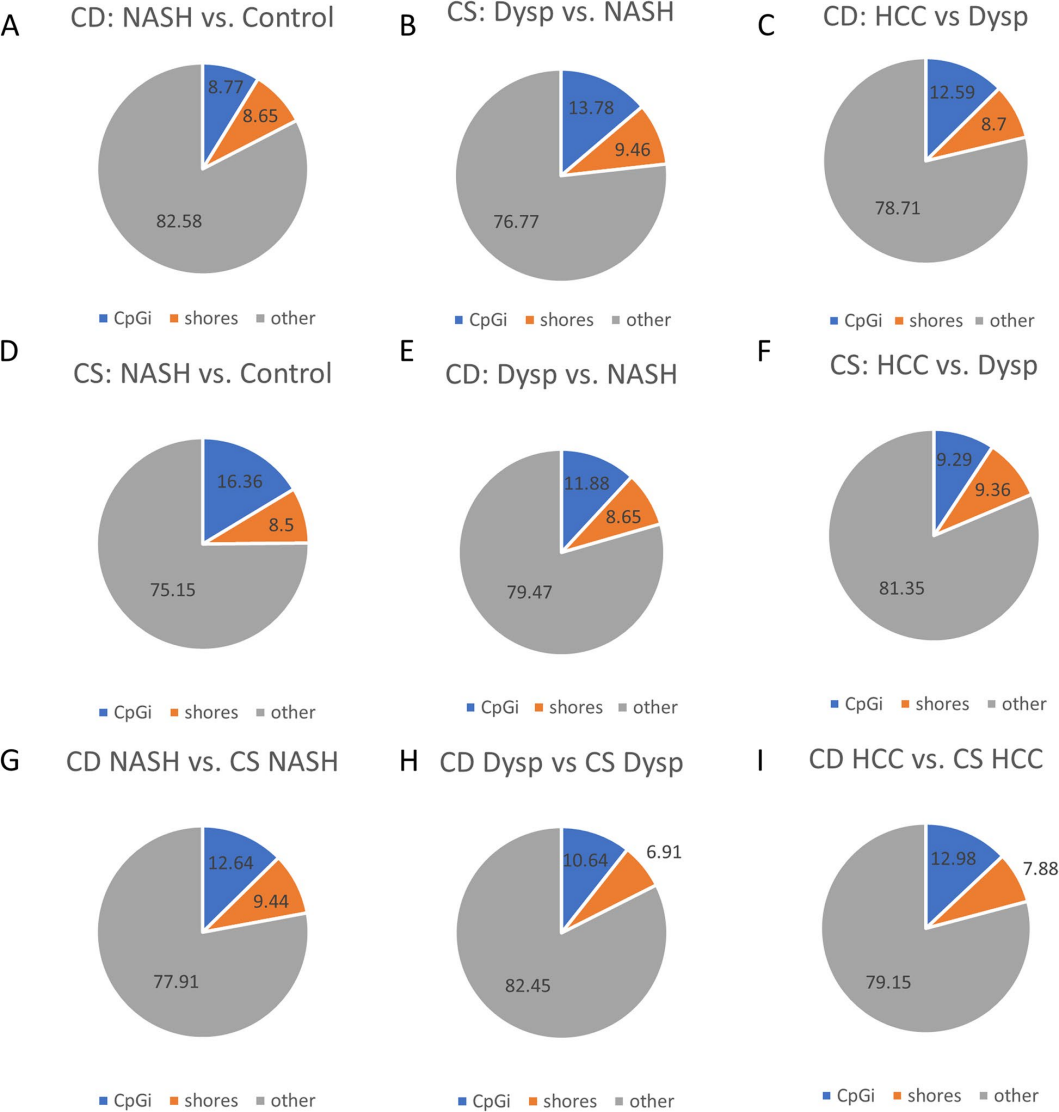
Percentages of CpG categories (islands, shores, or other) for significant differential DNA methylation loci. **A**. CD: NASH vs. Control. **B**. CD: Dysp vs. NASH **C**. CD: HCC vs. NASH. **D**. CS: NASH vs Control. **E**. CS: Dysp vs. NASH **F**. CS: HCC vs. Dysp. **G**. CD NASH vs. CS NASH. **H**. CD Dysp. vs. CS Dysp. **I**. CD HCC vs. CS HCC

Differentially methylated regions between each comparison (diet or disease stage) were categorized as occurring either in CpG islands, shores, or other regions (regions greater than 2 kb from CpG islands). Among all comparisons shown in Fig. 1, the majority of DMRs occurred in regions other than CpG islands and shores. In the choline deficient model, there were an increasing proportion of DMRs in CpG islands through the progression of HCC and decreasing DMRs in the other regions (Fig. 1A-C). In the choline supplemented model, there was a decreasing proportion of DMRs in CpG islands and an increasing proportion of DMRs in the other regions through the progression of HCC (Fig. 1D-F). No uniform pattern of DMRs by gene region was seen between the choline deficient and supplemented models at the NASH, dysplastic, and HCC stages.

**Fig. 2:**
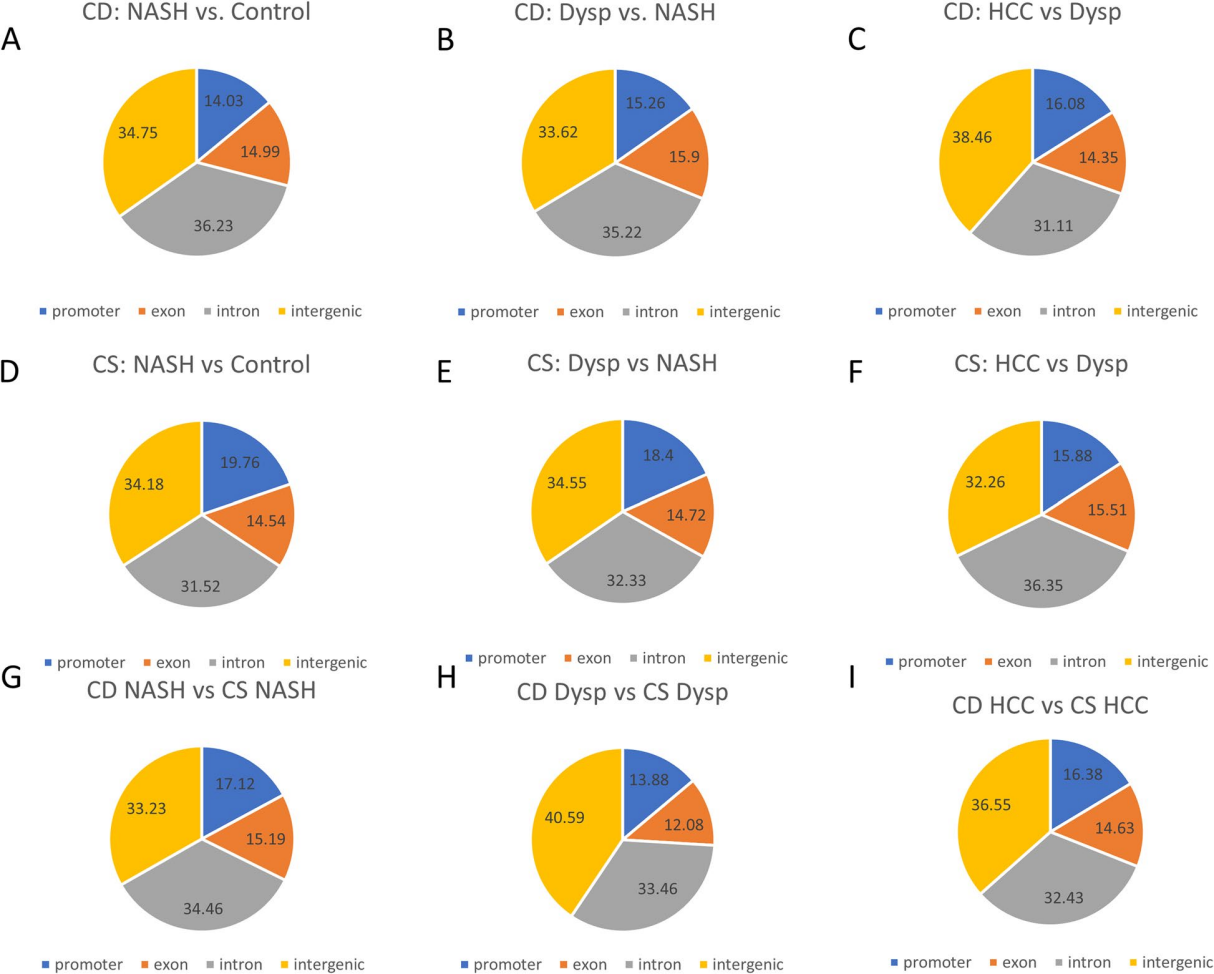
Percentages of gene structure categories for significant differential DNA methylation loci. **A**. CD: NASH vs. Control. **B**. CD: Dysp vs. NASH **C**. CD: HCC vs. NASH. **D**. CS: NASH vs Control. **E**. CS: Dysp vs. NASH **F**. CS: HCC vs. Dysp. **G**. CD NASH vs. CS NASH. **H**. CD Dysp vs. CS Dysp. **I**. CD HCC vs. CS HCC

The proportion of DMRs by methylation loci (promoter, exon, intron, and intergenic regions) are shown in Fig. 2. Similar patterns were observed across the progression of HCC in the choline deficient and supplemented models. Between the comparisons of the two diet models at each stage (Fig. 2G-I), the largest difference was seen in the proportion of DMRs in intergenic regions between the NASH and dysplastic stages, with the latter showing a larger proportion of DMRs in intergenic regions.

**Fig. 3:**
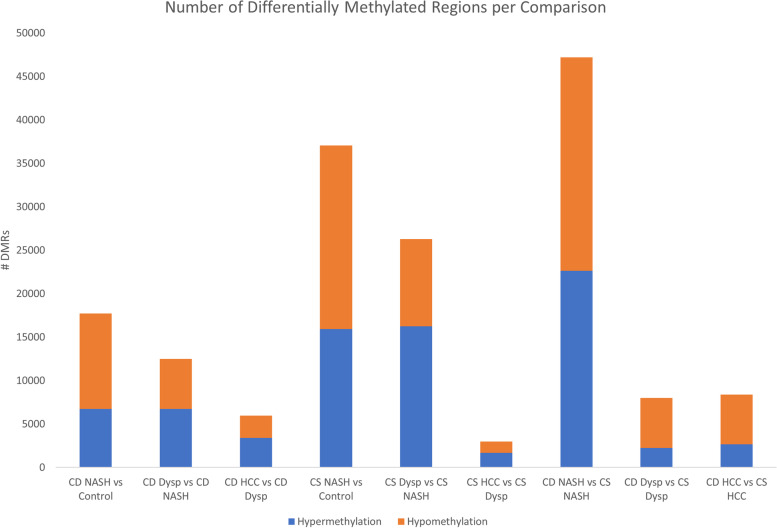
Number of differentially methylated regions by comparison

Figure 3 shows the number of differentially methylated regions per comparison, as well as the proportion of hypermethylated and hypomethylated regions per each comparison. In both diet models, the largest number of DMRs was seen between NASH and the controls; the lowest number of DMRs was seen between HCC and the dysplastic stage (Supplemental File 1). Among the disease stage progression, there was a higher number of DMRs among the choline supplemented model than the choline deficient model, except for the HCC vs Dysplastic Nodules comparison. The proportion of DMRs that were hypermethylated increased throughout the HCC progression in both models, even though the number of hypermethylated regions decreased with tumor progression. In the final three comparisons in Fig. 3 that compare the stages between the two diet models, the highest number of DMRs was seen at the NASH stage. Compared to the choline deficient model, there was a greater proportion of hypomethylated DMRs in the choline supplemented model in the NASH, dysplastic, and HCC stages.

### Genomic distribution of DMRs

**Fig. 4:**
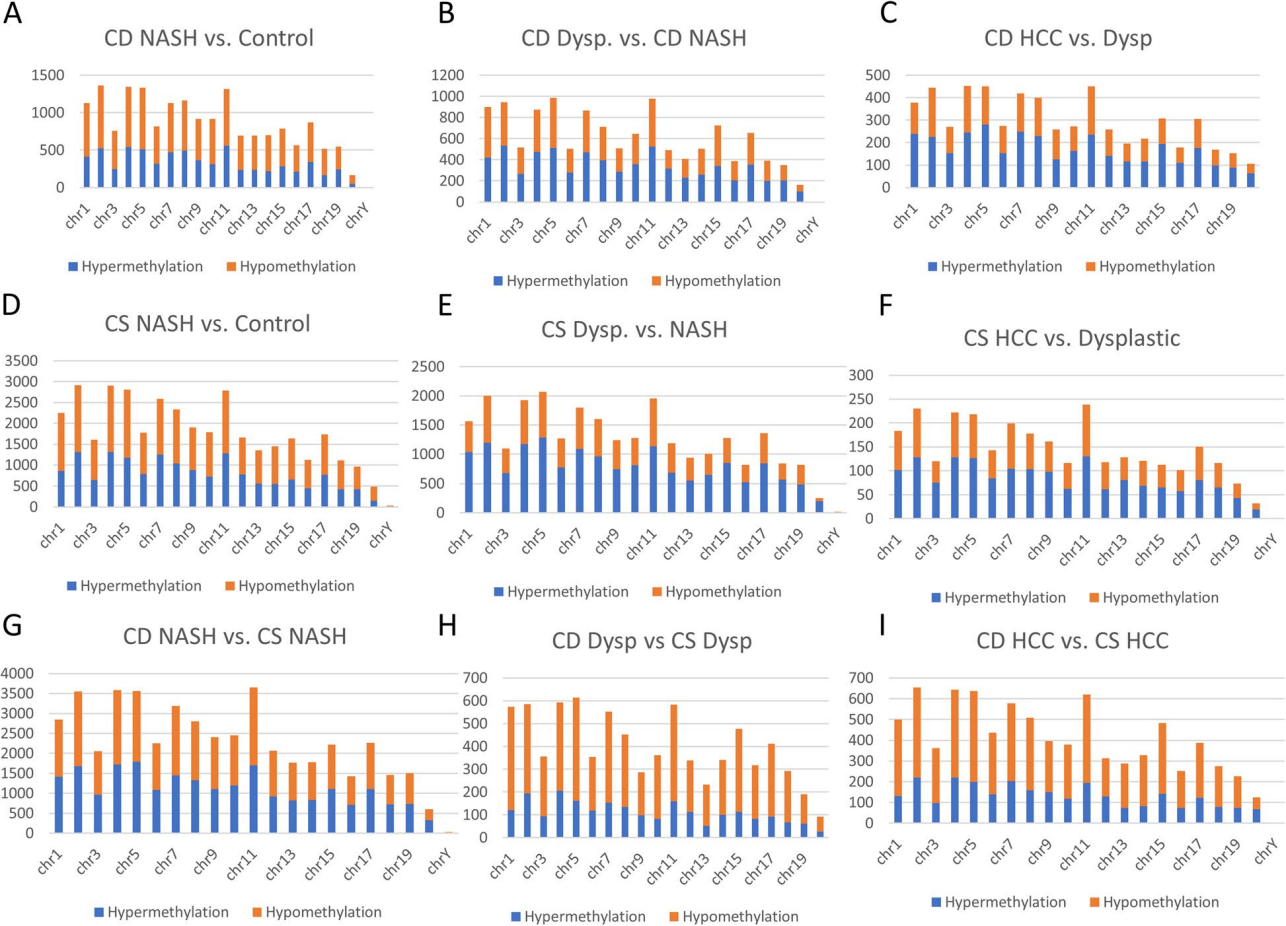
Number of differentially methylated regions per chromosome. **A**. CD: NASH vs. Control. **B**. CD: Dysp vs. NASH **C**. CD: HCC vs. NASH. **D**. CS: NASH vs Control. **E**. CS: Dysp vs. NASH **F**. CS: HCC vs. Dysp. **G**. CD NASH vs. CS NASH. **H**. CD Dysp vs. CS Dysp. **I**. CD HCC vs. CS HCC

There were higher numbers of differentially methylated regions in the progression of HCC in the choline-supplemented model (Fig. 4D-F, Supplemental File 1) compared to the choline-deficient model (Fig. 4A-C) for each chromosome. While the numbers of DMRs were different, the overall pattern per chromosome was similar in both diet models. The proportion of DMRs that were hypermethylated was higher in the choline-supplemented model. Comparing each stage of progression between the two models, there was a greater proportion of hypomethylation per chromosome in the dysplastic and HCC stages compared to NASH (Fig. 4G-I, Supplemental File 1).

### Genes potentially modified by hyper/hypomethylation in the progression of HCC

Comparing HCC and NASH in the CD and CS models, a greater proportion of DMRs were in CpG islands and promoter regions in the CS model; while there was significantly more DMRs in the CS model compared to the CD model, the proportion of DMRs per chromosome was similar (Supplemental Fig. 1). The top ten differentially hypermethylated and hypomethylated regions with the greatest percent methylation differences between CD HCC and NASH are presented in Table 1. The regions with the largest percent methylation difference were a mix of hypermethylated and hypomethylated regions; the majority were outside of CpG islands and shores and were found mainly in exons or introns. Within the CD model, several of the largest differentially methylated regions are in genes involved in lipid metabolism (PNPLA6 and LDLRAP1), transcription (CHCHD2), and Wnt signaling pathways (JADE1), and cell migration and binding (TMEM88b, MYO5b, FSNC1, and ZDHHC12) [11, 12]. The top ten differentially hypermethylated and hypomethylated regions between CS HCC and NASH are presented in Table 2. The percent methylation difference was larger among the hypermethylated regions. There was no overlap in the regions with the highest percent methylation difference between HCC and NASH in the choline deficient and supplemented models. DMRs were located mostly outside of CpG islands and shores and were found in introns and exons. The percent methylation differences between HCC and NASH were higher in the CS model (Table 2) than in the CD model (Table 1). Within the CS model, the significantly differentially methylated regions are found in genes involved in ion channels (GJA8, KCNQ5, and LRRC8D), cell cycle regulation (PTPRE and RNF123), and cell signaling pathways (RNF217), including Wnt signaling (SOSTDC1) [11, 12]. Four of the differentially methylated regions were in genes previously found to be associated with obesity, metabolic regulation, and glycemic control (LRRC8D, SOSTCD1, MORN3, and MCF2) [16–19].

**Table 1:** Top 10 hypermethylated and hypomethylated regions between HCC and NASH in the choline deficient model

	Chromosome	Start	End	Q-value	Methylation difference (%)	Gene	CpG Category	Gene Structure
Hypermethylation	Chr 8	3517616	3517616	1.65E-48	76.2212	PNPLA6	Shore	Exon
	Chr 17	32105855	32105855	8.63E-47	68.636	–	Other	–
	Chr 11	99645764	99645764	5.30E-74	67.33949	KRTAP4–7	Other	–
	Chr 5	129557719	129557719	4.11E-59	65.9518	CHCHD2	Other	Exon
	Chr 5	142945496	142945496	1.39E-55	65.23347	FSCN1	Other	–
	Chr 3	41581453	41581453	2.32E-44	64.13043	JADE1	Other	Exon
	Chr 9	121792412	121792412	5.80E-53	62.74613	–	Other	Intron
	Chr 4	155781218	155781218	4.83E-39	62.30984	TMEM88B	Other	Intron
	Chr 6	31666012	31666012	1.21E-81	62.05207	Gm13848	Other	Exon
	Chr 18	82756567	82756567	7.26E-80	61.81989	–	Other	Intergenic
Hypomethylation	Chr 2	30100458	30100458	3.98E-61	−68.875	ZDHHC12	Other	Intron
	Chr 2	170791632	170791632	8.07E-49	−67.2973	–	Other	Intron
	Chr 10	69846110	69846110	8.28E-53	−66.5238	GM33416	Other	Intron
	Chr 9	57968665	57968665	1.39E-36	−63.029	MYO5B	Other	Intron
	Chr 4	110051897	110051897	5.55E-47	−62.4214	DMRTA2	Shore	–
	Chr 16	97823834	97823834	1.62E-34	−61.5291	–	Other	Intron
	Chr 4	134744349	134744349	5.94E-31	−60.3271	LDLRAP1	Other	Exon
	Chr 7	36873554	36873554	7.09E-53	−59.9929	–	Other	–
	Chr 7	42750002	42750002	3.33E-34	−59.3331	DOCK3	Other	Exon
	Chr 5	74960682	74960682	4.97E-51	−59.0323	Gm6116	Other	Intron

*Abbreviation: Chr* Chromosome

**Table 2:** Top 10 hypermethylated and hypomethylated regions between HCC and NASH in the choline supplemented model

	Chromosome	Start	End	Q-value	Methylation difference (%)	Gene	CpG Category	Gene Structure
Hypermethylation	Chr 10	31860051	31860051	5.08E-36	100	RNF217	Other	Intron
	Chr 3	96919939	96919939	6.65E-59	98	GJA8	Other	Exon
	Chr 17	54643532	54643532	2.75E-37	97.71429	Gm32055	Other	–
	Chr 1	21988549	21988549	3.91E-54	96.51515	KCNQ5	Other	Intron
	Chr 5	142385284	142385284	2.78E-42	95.35104	PTPRE	Other	–
	Chr 2	123549054	123549054	1.02E-29	95.26627	MORN3	Other	–
	Chr 3	55348282	55348282	1.08E-31	93.17359	Gm40051	Other	Intron
	Chr 13	51672327	51672327	5.01E-42	91.07143	SECISBP2	Other	Intron
	Chr X	60093579	60093579	4.50E-29	90.47619	MCF2	Other	Intron
	Chr 16	29868324	29868324	8.94E-38	89.09953	RNF123	Other	–
Hypomethylation	Chr 5	36228842	36228842	1.50E-57	−87.4512	PSAPL1	Other	Intron
	Chr 4	98726981	98726981	1.18E-39	−87.4396	L1TD1	Other	Intron
	Chr 4	82537544	82537544	7.33E-64	−87.2659	–	Other	Intron
	Chr 7	108951080	108951080	1.18E-26	−86.2069	Gm39067	Island	Intron
	Chr 11	82930668	82930668	8.16E-37	−86.1035	UNC45BOS	Shore	Exon
	Chr 5	128527390	128527390	1.68E-27	−85.9649	–	Other	–
	Chr 5	105759229	105759229	1.97E-27	−84.8837	LRRC8D	Other	Intron
	Chr 12	25240371	25240371	6.01E-20	−84.3137	–	Other	Intron
	Chr 2	172863024	172863024	6.24E-28	−83.871	–	Other	Intron
	Chr 12	36317937	36317937	8.35E-39	−83.8572	SOSTDC1	Shore	Exon

*Abbreviation*: *Chr* Chromosome

### Differences in methylation patterns of hepatocellular carcinoma in lean and obese mice

Table 3 shows the top 10 differentially hypermethylated and hypomethylated regions between HCC in the CD model and HCC in the CS model. All but one of the top 10 overall regions with the highest percent difference in methylation were hypomethylation, indicating that there was a greater degree of methylation in the choline supplemented model HCC compared to the choline deficient model. Of the hypermethylated regions, half were in CpG islands and shores. Differential methylation was found mostly in exons and introns. The corresponding genes are involved in physiological process including cell signaling pathways, catabolic processes, and protein translation [11, 12]. Five hypermethylated regions were in genes previously associated with cancer (TRAP1, SLC38A3, CHRM1, EDN2, and PROX1), while six hypomethylated regions were in genes previously associated with cancer (NUMBL, ALDH1B1, FTCD, FASTKD2, FAM96A, and ARHGAP15) [20–30]. Five differentially methylated regions were in genes previously found to be associated with obesity and altered metabolic states (TRAP1, SLC38A3, PROX1, ALDH1B1, and FAM96A) [31–35].

**Table 3:** Top 10 hypermethylated and hypomethylated regions between choline deficient HCC and choline supplemented HCC

	Chromosome	Start	End	Q-value	Methylation difference (%)	Gene	CpG Category	Gene Structure
Hypermethylation	Chr 7	27658325	27658325	5.06E-41	68.16218	TTC9B	Island	Exon
	Chr 5	110363759	110363759	1.58E-41	57.24965	LCROL1	Island	Exon
	Chr 1	42741736	42741736	1.39E-86	56.99229	LOC108167622	Other	Intron
	Chr 16	4078449	4078449	8.78E-54	55.9661	TRAP1	Shore	–
	Chr 7	107210287	107210287	4.31E-25	54.50725	RBMXL2	Island	Exon
	Chr 9	107669835	107669835	3.47E-63	54.2572	SLC38A3	Other	Intron
	Chr 19	8679239	8679239	1.30E-35	54.08986	CHRM1	Other	Exon
	Chr 4	120124981	120124981	7.45E-29	54.015	EDN2	Other	Exon
	Chr 7	107210301	107210301	1.45E-24	53.93071	RBMXL2	Island	Exon
	Chr 1	190139252	190139252	8.04E-63	53.61003	PROX1	Other	Intron
Hypomethylation	Chr 9	121076534	121076534	2.87E-43	−66.7969	OLFR843	Other	Intron
	Chr 7	27257369	27257369	6.56E-67	−62.1354	NUMBL	Shore	Exon
	Chr 10	76634403	76634403	3.22E-65	−61.6133	–	Other	Intron
	Chr 4	45785050	45785050	1.68E-54	−60.7105	ALDH1B1	Other	–
	Chr 10	76584578	76584578	4.26E-46	−59.9492	FTCD	Other	Exon
	Chr 12	74283740	74283740	5.55E-38	−59.9486	FASTKD2	Shore	Intron
	Chr 10	73300112	73300112	2.99E-43	−59.8009	FAM96A	Other	Intron
	Chr 9	46986808	46986808	1.95E-46	−59.3403	GM4791	Other	–
	Chr 2	44162442	44162442	5.78E-45	−59.2628	ARHGAP15	Other	Intron
	Chr 7	37356388	37356388	3.96E-45	−58.4701	–	Other	–

*Abbreviation*: *Chr* Chromosome

### Functional analyses

Gene Ontology enrichment analysis was performed on the genes indicated as having a 25% or greater methylation difference in HCC versus NASH in both the choline deficient and choline supplemented models. The DMRs in both comparisons shared some functions, including transcription activation and DNA binding in transcription (Table 4). Functions specific to the DMRs of the choline deficient model of HCC progression included hormone binding and calcium ion binding. Functions specific to the DMRs of the choline supplemented model included neuropeptide receptor activity, voltage-gated potassium channel activity, transcription repressor activity, and signaling receptor activator activity. Additionally, of the hypomethylated regions in HCC versus NASH, the molecular functions of Wnt-protein binding and frizzled binding were found to be enriched in the choline supplemented model, but not the choline deficient model (Table 5). These different functional activities of differentially methylated regions may be involved in pathways associated with the progression of HCC from NASH in the context of lean and obese mice.

**Table 4:** Top statistically significant Gene Ontology molecular functions

Model	Molecular Function	Fold Enrichment
CD HCC vs. NASH	Hormone binding	3.05
	DNA-binding transcription activator activity, RNA polymerase II-specific	1.88
	RNA polymerase II cis-regulatory region sequence-specific DNA binding	1.72
	Calcium ion binding	1.57
CS HCC vs. NASH	Neuropeptide receptor activity	2.76
	Voltage-gated potassium channel activity	2.03
	DNA-binding transcription activator activity, RNA polymerase II-specific	1.61
	DNA binding transcription repressor activity, RNA polymerase-II specific	1.5
	RNA polymerase II cis-regulatory region sequence-specific DNA binding	1.49
	Signaling receptor activator activity	1.39

Top statistically significant Gene Ontology molecular functions revealed by enrichment analysis between HCC and NASH in the choline deficient and choline supplemented mice

**Table 5:** Enriched molecular functions in hypomethylated regions between HCC and NASH

Model	Molecular Function	Fold Enrichment
CD HCC vs. NASH	DNA-binding transcription activator activity, RNA polymerase II-specific	2.14
	RNA polymerase II cis-regulatory region sequence-specific DNA binding	1.82
	Protein binding	1.17
CS HCC vs. NASH	Wnt-protein binding	4.20
	Frizzled binding	3.17
	DNA-binding transcription activator activity, RNA polymerase II-specific	2.21
	DNA-binding transcription repressor activity, RNA polymerase II-specific	2.07
	RNA polymerase II cis-regulatory region sequence-specific DNA binding	2.00
	Receptor ligand activity	1.52
	Cation binding	1.18

Top statistically significant Gene Ontology molecular functions revealed by enrichment analysis of hypomethylated regions between HCC and NASH in the choline deficient and choline supplemented mice

## Discussion

**Fig. 5:**
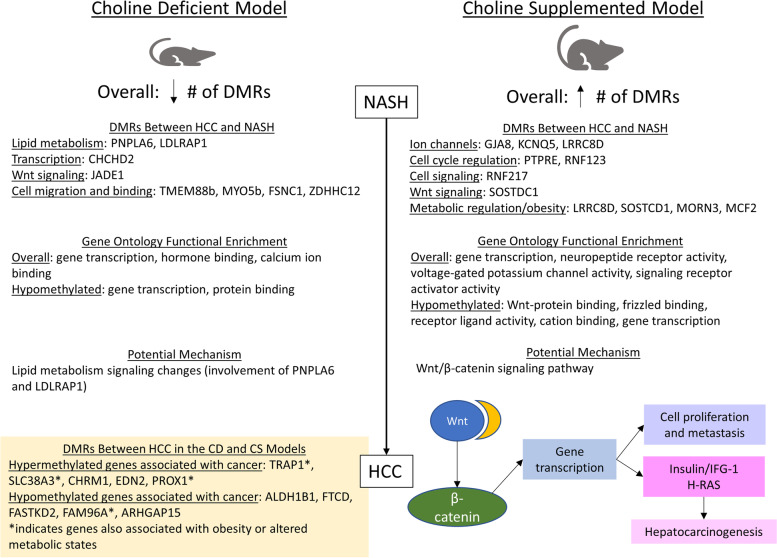
Summary of differential methylation in the CD and CS models. A summary of the differential methylation patterns between the choline deficient and choline supplemented models, differentially methylated genes and functions, and potential implications

In this work, we demonstrated that significant differential methylation exists in the progression of NASH-HCC in the context of obesity (through choline supplementation) and non-obesity (through choline deficiency) in mice fed a high fat/fructose/cholesterol diet (Fig. 5). Larger numbers of DMRs were found between HCC and NASH in the obese mice (41,979) compared to the non-obese mice (11,104). In both models, the largest number of DMRs were seen at the beginning stages of disease progression (in NASH and dysplasia) and the proportion of DMRs that were hypermethylated increased with progression. DNA methylation has previously been implicated in carcinogenesis; previous studies have identified differential methylation in NASH-related HCC in both mice and humans [36, 37]. Global hypomethylation is common in carcinogenesis, with numerous methylation changes occurring early in tumor progression [38]. The increased number of DMRs and hypermethylation in the obese mice may be driven by dietary choline, which is involved in one-carbon metabolism in methylation [39]. Additionally, our previous work found that mice with HCC in the context of obesity had higher plasma fasting glucose and cholesterol levels than lean mice with HCC [40]; differences in glucose levels may contribute to the differential methylation patterns identified, which is in line with previous work that found glucose levels were associated with CpG methylation levels [41].

In the choline deficient model, significant hypermethylation of CHCHD2 (within an exon) and FSCN1 was seen. Overexpression of both of these genes has been found to be associated with HCC and found to be an indicator of poor prognosis [42–44]. Our previous work identified faster progression of HCC and worse survival in the choline deficient model; the role of these genes in carcinogenesis may depend on the stage of progression and site of methylation. Significant differential methylation was also found in exons of two genes involved in lipid metabolism, PNPLA6 (within a CpG shore) and LDLRAP1 [11].

In the choline supplemented model, significant hypermethylation of RNF217 was found, which has also been seen with alcohol-related HCC in humans [45]. RNF217 is involved in process of ubiquitination [11]. Differential methylation was also seen in genes previously identified as tumor suppressor genes and oncogenes, including KCNQ5, PTPRE, and SOSTDC1 [46–48]. Additionally, hypermethylation was found in GJA8 (within an exon) and hypomethylation was found in PSAPL1 and LRRC8D (within introns); these three genes have all been found to be associated with hepatocarcinogenesis [49–51]. Increased methylation of MORN3 and decreased methylation of LRRC8D in the context of obesity is consistent with previous studies [16, 18]. Hypermethylation of MCF2, a proto-oncogene involved in Rho protein signal transduction, was also seen in obese patients with breast cancer [11, 19].

Comparing HCC in the choline deficient model and choline supplemented model, significant methylation differences were seen in 11 genes previously associated with cancer (hypermethylation of TRAP1, SLC38A3, CHRM1, EDN2, and PROX1; hypomethylation of ALDH1B1, FTCD, FASTKD2, FAM96A, and ARHGAP15). Of these genes, four (TRAP1, SLC38A3, PROX1, and FAM96A) have also been associated with obesity and altered metabolic states, indicating that they may be implicated in differential progression of lean versus obese NASH-HCC (Fig. 5).

No overlap exists in the regions with the highest methylation difference between HCC and NASH in the lean and obese models, indicating that differential methylation may act through different mechanisms to promote carcinogenesis in the two models. Gene ontology enrichment analysis on the DMRs between HCC and NASH in the two models revealed some shared molecular functions, including functions related to gene transcription. The choline deficient model had DMRs in genes involved with hormone binding and calcium ion binding, while the choline supplemented model had DMRs in genes involved in neuropeptide receptor activity, voltage-gated potassium channel activity, and signaling receptor activator activity. These signaling pathway molecular functions may represent potential mechanisms of HCC progression in the two models. Calcium signaling has previously been found to be enriched in NAFLD-associated HCC [52].

Looking at the functional enrichment of hypomethylated regions between HCC and NASH, which would result in overexpression of the associated genes, several important differences were seen between the two models. In the choline supplemented model, significant hypomethylation was seen in genes involved in Wnt-protein binding and frizzled binding, as well as receptor ligand activity and cation binding, which were not seen in the choline deficient model. Additionally, one gene in Table 2, SOSTDC1, was found to be hypomethylated (within a CpG shore of an exon); SOSTDC1 is involved in enhancing Wnt signaling pathways [11].

The Wnt/β-catenin signaling pathway has been implicated in several mechanisms of hepatocarcinogenesis, including growth, survival, and migration [53]. In the canonical Wnt signaling pathway, Wnt interacts with frizzled receptors and activates intracellular signaling pathways leading to the stabilization of β-catenin, which can then enter the nucleus and influence transcription of target genes [54, 55]. β-catenin may regulate the transcription of genes involved in proliferation and metastasis and may also interact with other oncogenic pathways such as insulin/IFG-1 and H-RAS to influence pathogenesis (Fig. 5) [53, 55]. Mutations in β-catenin have been found in a large proportion of liver tumors [55]. Additionally, β-catenin has been found to be involved in changing the tumor-immune microenvironment in NAFLD-associated HCC [56]. Hypomethylation of genes involved in Wnt/β-catenin signaling pathways likely contributes to the development and progression of HCC in the context of obesity. Functional enrichment of Wnt/ β-catenin was not seen in the model of lean NASH-HCC, indicating that direct involvement of the Wnt signaling pathways through methylation changes may be unique to the choline supplemented model. In the choline deficient model, differential methylation in exons of genes involved in lipid metabolism (PNPLA6 and LDLRAP1) may alter signaling pathways leading to the progression of HCC. Thus, tumor progression may involve lipid metabolism signaling changes in the lean NASH-HCC model. Differentially methylated regions may be targets for emerging epigenetic cancer therapeutics; additional work is needed to test the study results in humans and examine the effect of differential methylation on the effectiveness of available therapeutics [57]. Further research is needed to understand the underlying mechanisms through which differentially methylated genes drive NASH-HCC development, and progression and to examine differentially methylated regions in the progression of NASH-HCC in humans.

## Conclusions

A large number of differentially methylated regions are seen in the progression of NASH-HCC in both lean and obese mice; differential methylation is also seen between the stages of progression in the two models. With methylation differences seen in both mice and humans, further studies could be conducted using the obese and lean mice models to elucidate the mechanisms of progression of NASH-HCC in the context of obesity and normal weight. Additionally, differentially methylated regions may be able to serve as biomarkers for cancer progression or potential therapeutics, highlighting the importance of the study of epigenetic changes in hepatocarcinogenesis.

## Supplementary Information

**Additional file 1: Supplemental file 1.** Absolute numbers and proportions of comparisons by comparison and chromosome. Description: Absolute number of hypermethylated and hypomethylated regions, totals, and proportions of between each comparison and by chromosome number in the choline supplemented, choline deficient, and control models.

**Additional file 2: Supplemental Fig. 1.** Comparison of HCC vs. NASH in choline supplemented and choline deficient models. Description: Percentages of CpG categories (islands, shores, or other) and gene structure categories for significant differential DNA methylation loci, number of DMRs per comparison, and number of DMRs per chromosome. A. Percentages of CpG categories for CD: HCC vs. NASH Percentages of B. CpG categories for CS: HCC vs. NASH. C. Percentages of gene structure categories for CD: HCC vs. NASH D. Percentages of gene structure categories for CS: HCC vs. NASH. E. Number of differentially methylated regions by comparison. F. Number of DMRs per chromosome comparing CD: HCC vs. NASH. G. Number of DMRs per chromosome comparing CS: HCC vs. NASH.

## Data Availability

The datasets used and/or analyzed during the current study are available from the corresponding author on reasonable request.

## Abbreviations

NAFLD: Non-alcoholic liver disease
NASH: Non-alcoholic steatohepatitis
HCC: Hepatocellular carcinoma
CD: Choline deficient
CS: Choline supplemented
HFFC: High trans-fat, fructose, and cholesterol
RRBS: Reduced representation bisulfite sequencing
DMR: Differentially methylated region
