# Supplementary figures and images for: Differential methylation patterns in lean and obese non-alcoholic steatohepatitis-associated hepatocellular carcinoma
Source: BMC Cancer. 2022 Dec 6;22:1276. doi: 10.1186/s12885-022-10389-7 (PMC9727966; doi:10.1186/s12885-022-10389-7)

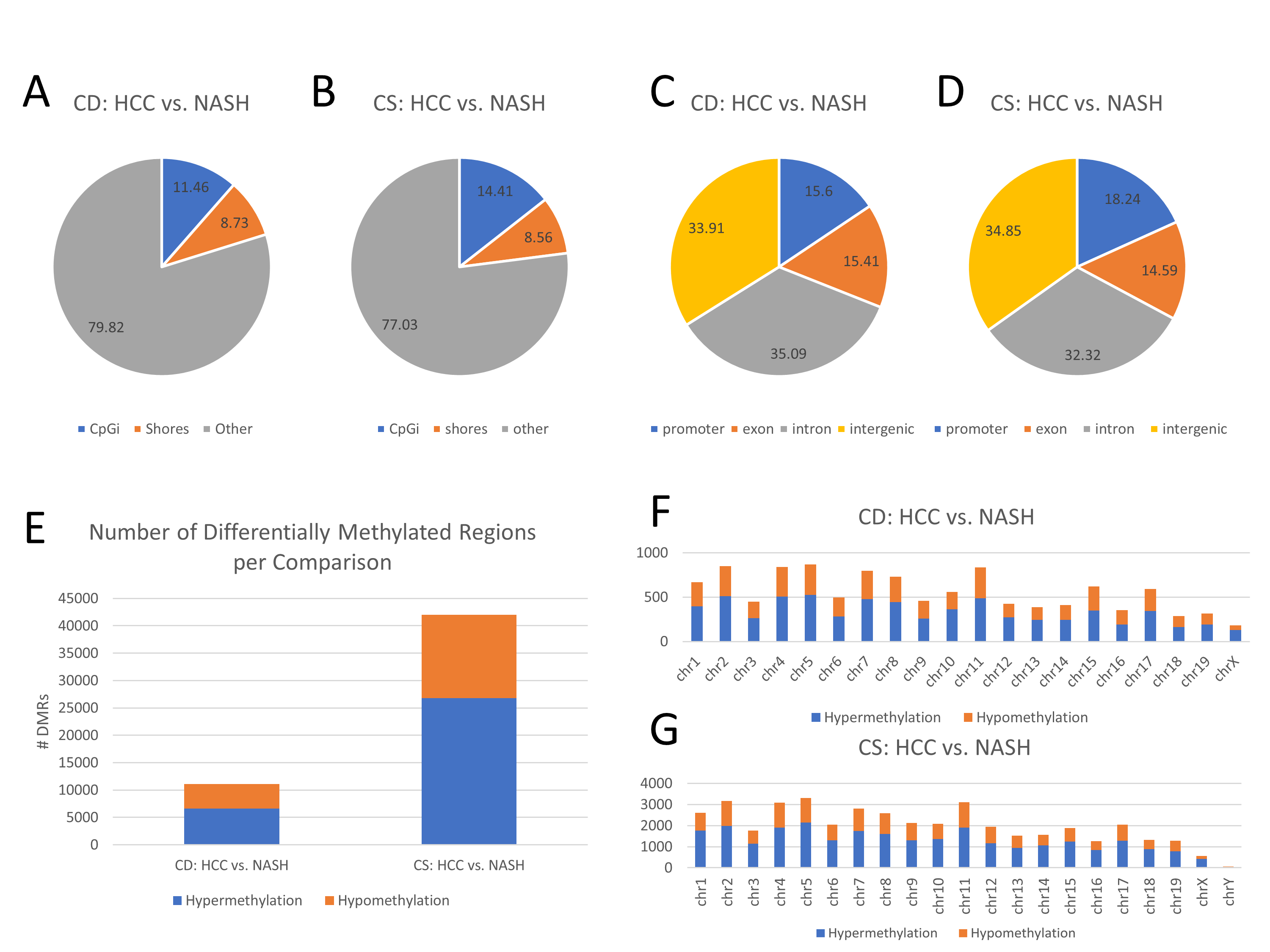

Supplement: Supplementary file 2 — Additional file 2: Supplemental Fig. 1. Comparison of HCC vs. NASH in choline supplemented and choline deficient models. Description: Percentages of CpG categories (islands, shores, or other) and gene structure categories for significant differential DNA methylation loci, number of DMRs per comparison, and number of DMRs per chromosome. A. Percentages of CpG categories for CD: HCC vs. NASH Percentages of B. CpG categories for CS: HCC vs. NASH. C. Percentages of gene structure categories for CD: HCC vs. NASH D. Percentages of gene structure categories for CS: HCC vs. NASH. E. Number of differentially methylated regions by comparison. F. Number of DMRs per chromosome comparing CD: HCC vs. NASH. G. Number of DMRs per chromosome comparing CS: HCC vs. NASH. [file 12885_2022_10389_MOESM2_ESM.tif]
